# From helicobacter pylori to glucose metabolism: can DOB values serve as a predictive marker?

**DOI:** 10.3389/fmed.2025.1612456

**Published:** 2025-08-13

**Authors:** Yu Zhou, Jie Xu, Zhigang Huang, Daoxing He, Hui Duan, Yating Shi, Zhenjie Wang, Zhaoyi Chen

**Affiliations:** ^1^Department of Gastroenterology, Xuancheng People’s Hospital, Affiliated Xuancheng Hospital Wannan Medical College, Xuancheng, Anhui, China; ^2^Department of Electrophysiology, Huai’an No.3 People’s Hospital, Huai’an, Jiangsu, China

**Keywords:** *Helicobacter pylori*, fasting blood glucose, urea breath test, delta-over-baseline, metabolism abnormalities

## Abstract

**Background:**

*Helicobacter pylori* infection and abnormal glucose metabolism are prevalent, interconnected contributors to chronic disease. Although metabolic changes have been studied in infected individuals, the independent association between the delta-over-baseline (DOB) value of the ^13^C-urea breath test and fasting blood glucose (FBG) remains unclear. We investigated whether DOB could predict abnormal FBG in adults receiving routine health examinations.

**Objectives:**

To assess the association between *H. pylori* infection and metabolic abnormalities, and to evaluate the predictive utility of the DOB value for glycemic abnormalities.

**Methods:**

In this retrospective study, 594 patients underwent both the ^13^C-UBT and metabolic parameter assessments. Patients were stratified by DOB values, and metabolic abnormalities were defined by predefined criteria. Logistic regression analyzed the relationship between *H. pylori* status and metabolic parameters, adjusting for confounders. A restricted cubic spline (RCS) model and receiver operating characteristic (ROC) curve assessed non-linear associations and diagnostic performance of DOB.

**Results:**

Compared with the *H. pylori*-negative group, the positive group exhibited significantly higher triglyceride (1.667 ± 1.173 vs. 1.447 ± 0.954 mmol/L; *p* = 0.020) and FBG levels (5.655 ± 1.704 vs. 5.363 ± 1.028 mmol/L; *p* = 0.024). In multivariable models, *H. pylori* infection was independently associated with abnormal FBG (OR 2.10; 95% CI 1.30–3.40; *p* = 0.003) but not with TG abnormalities. The DOB value showed modest overall discriminatory ability for abnormal FBG (AUC = 0.590), with enhanced performance in participants < 40 years (AUC = 0.721).

**Conclusion:**

*H. pylori* infection is associated with higher fasting glucose and triglyceride levels, and the ^13^C-UBT DOB value showed modest predictive ability for glycemic abnormalities—especially in adults under 40 (AUC = 0.721). DOB may serve as an adjunct risk-stratification marker in younger populations. However, the single-center, cross-sectional design and lack of lifestyle and mechanistic biomarker data limit causal inference. Prospective multicenter cohort studies with serial UBT, clinical (diet, medications, exercise, socioeconomic factors) and biomarker (cytokines, GLP-1) measurements are needed to validate these findings.

## 1 Introduction

*Helicobacter pylori* (*H. pylori*) is one of the most prevalent chronic infections worldwide, widely colonizing the gastric mucosa and closely associated with chronic gastritis, peptic ulcer disease, and gastric cancer (1). Emerging evidence indicates that *Helicobacter pylori* infection is associated with metabolic disturbances beyond the gastrointestinal tract, including insulin resistance, dyslipidemia, and non-alcoholic fatty liver disease (NAFLD) (2).

Dyslipidemia is a key risk factor for cardiovascular disease, with elevated triglyceride (TG) levels being closely linked to an increased risk of atherosclerosis (3). At the same time, abnormal blood glucose (BG) levels are an integral component of metabolic syndrome, and the coexistence of dyslipidemia and hyperglycemia further elevates cardiovascular risk. However, studies investigating the relationship between *H. pylori* infection and lipid as well as glucose metabolism have yielded inconsistent results (4, 5). Some researchers have reported that *H. pylori* infection is associated with altered blood lipid and glucose profiles—potentially linked to chronic inflammation, shifts in gut microbiota composition, or endocrine modulation—while other studies have observed no significant associations or have produced conflicting results. These discrepancies may be attributed to differences in study populations, sample sizes, and *H. pylori* detection methods.

Moreover, the ^13^C-urea breath test (^13^C-UBT) is a widely used non-invasive method for detecting *H. pylori* infection, with the delta-over-baseline (DOB) value reflecting bacterial activity and load. Previous studies have demonstrated that the DOB value correlates with the degree of gastric inflammation (6), Nonetheless, systematic investigations into the potential utility of the DOB value for predicting abnormalities in blood glucose and lipid levels are still lacking. Therefore, the present study aims to explore the relationship between *H. pylori* infection and blood lipid and glucose levels, and to further evaluate the application prospects of the DOB value in predicting metabolic abnormalities. This research is expected to offer new insights and evidence to support clinical practice.

## 2 Materials and methods

### 2.1 Study subjects

This retrospective cross-sectional study enrolled 594 patients who underwent the ^13^C-urea breath test (^13^C-UBT) at our institution between July 2023 and December 2024. Inclusion criteria were: age > 18 years and availability of complete clinical records. Exclusion criteria included: (1). a previous diagnosis of diabetes mellitus or dyslipidemia (e.g., hyperlipidemia); (2). the presence of thyroid dysfunction, chronic liver disease, nephrotic syndrome, malignancy, or autoimmune disorders; (3). long-term use of medications that may affect lipid metabolism (e.g., statins or fibrates). Based on the ^13^C-UBT results, subjects were classified into an *H. pylori*-positive group (DOB value ≥ 4‰) and an *H. pylori*-negative group (DOB value < 4‰). In addition, clinical data including lipid profiles [total cholesterol (TC), triglycerides (TG), high-density lipoprotein cholesterol (HDL-C), low-density lipoprotein cholesterol (LDL-C)], fasting blood glucose (FBG), age, gender, body mass index (BMI), smoking and drinking status, and history of chronic diseases were collected.

### 2.2 Detection methods

A standardized protocol was employed to assess *H. pylori* infection and metabolic parameters.

#### 2.2.1 *H. pylori* detection

All patients were tested for *H. pylori* using a ^13^C-urea breath test instrument provided by a commercial supplier. The procedure was as follows:

   1.  After fasting for at least 2 h, a baseline breath sample was collected at 0 min and securely sealed.
   2.  The patient ingested a capsule containing 75 mg of ^13^C-urea, with timing initiated immediately thereafter.
   3.  A second breath sample was collected 30 min post-ingestion.
   4.  Both samples were analyzed using an isotope ratio mass spectrometer; a calculated DOB value ≥ 4‰ was considered indicative of *H. pylori* positivity.

#### 2.2.2 Metabolic parameter detection

Lipid profiles were measured via enzymatic methods. Total cholesterol (TC) was determined using the CHOD-PAP method, with levels > 5.2 mmol/L deemed abnormal; triglycerides (TG) were measured using the GPO-PAP method, with abnormal values defined as > 1.7 mmol/L; LDL-C was quantified by a detergent-based clearance method, with a threshold of > 3.1 mmol/L; and HDL-C was measured using a selective inhibition method, with abnormal values defined as > 1.96 mmol/L. Fasting blood glucose (FBG) was measured using the glucose oxidase method, with levels > 6.1 mmol/L considered abnormal.

### 2.3 Statistical analysis

Data were analyzed using SPSS version 25.0. Continuous variables with a normal distribution (TC, TG, HDL-C, LDL-C, FBG, BMI) were expressed as mean ± standard deviation, and intergroup differences were evaluated using independent-sample *t*-tests. Categorical variables (gender, smoking history, drinking history, and chronic disease history) were expressed as frequencies and percentages, with group differences assessed via the chi-square test. We prespecified a subgroup analysis by BMI category according to Chinese adult guidelines (WS/T 428–2013), using a cutoff of 24 kg/m^2^ to define overweight/obesity. Within each stratum (< 24 vs. ≥ 24), we conducted multivariable logistic regression including age, sex, and *H. pylori* status to assess whether the association between *H. pylori* infection and abnormal FBG differed by BMI.

#### 2.3.1 Independent Association Analysis

Binary logistic regression models were used to explore the independent associations between *H. pylori* infection and abnormalities in TG (> 1.7 mmol/L) and FBG (> 6.1 mmol/L), adjusting for potential confounders such as age, gender, and BMI.

#### 2.3.2 Diagnostic performance evaluation

A restricted cubic spline (RCS) model was used to explore the non-linear relationship between DOB and blood glucose abnormalities; the predictive ability of the DOB value for glycemic metabolic abnormality was assessed using receiver operating characteristic (ROC) curve analysis. The area under the curve (AUC) and its 95% confidence interval (95% CI) were calculated, and the optimal diagnostic cut-off value was determined using the Youden index.

## 3 Results

A total of 594 subjects were included in the study, with a mean age of 54.45 ± 11.96 years, and 47.5% (282/594) were male. The overall *H. pylori* infection rate was 35.4% (210/594), with a significantly higher infection rate in males compared to females (41.84% vs. 29.49%, χ^2^ = 9.896, *p* = 0.002).

No significant difference in age was observed between the two groups (*p* = 0.092). However, the *H. pylori*-positive group had a significantly higher BMI compared to the negative group (*p* = 0.015). Regarding lifestyle factors, the *H. pylori*-positive group demonstrated significantly higher rates of smoking (*p* = 0.004) and drinking (*p* = 0.003) compared to the *H. pylori*-negative group, while there were no significant differences in the prevalence of hypertension, coronary heart disease, or cerebrovascular disease (*p* > 0.05) (Table 1).

**TABLE 1:** summarizes the baseline characteristics of the study participants, stratified by *H. pylori* status.

Variable	*H. pylori*-positive (*n* = 210)	*H. pylori*-negative (*n* = 384)	x^2^/t	*P* -value
Gender (male)	(*n* = 118)	(*n* = 164)	9.896	0.002
Age	53.330 ± 11.848	55.060 ± 11.990	1.688	0.092
Smoking	(*n* = 41)	(*n* = 42)	8.326	0.004
Alcohol drinking	(*n* = 26)	(*n* = 21)	8.902	0.003
Hypertension	(*n* = 75)	(*n* = 116)	1.887	0.17
Coronary heart disease	(*n* = 9)	(*n* = 9)	1.742	0.187
Cerebrovascular disease	(*n* = 8)	(*n* = 12)	0.536	0.464
BMI	23.826 ± 3.307	23.149 ± 3.103	−2.437	0.015

**FIGURE 1:**
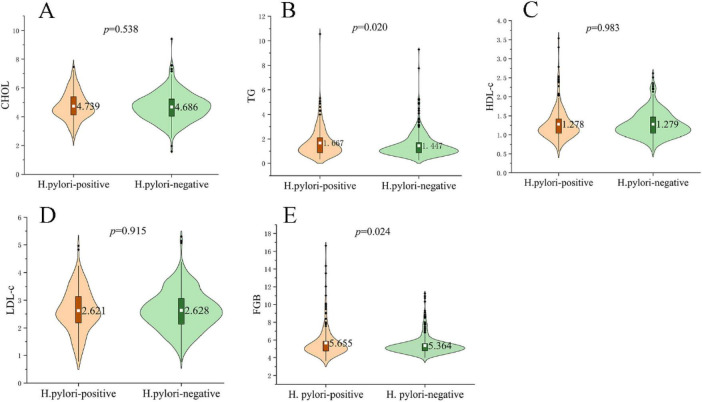
Metabolic profiles in *H. pylori*–positive and negative subjects. Violin plots show the distribution of **(A)** total cholesterol (CHOL), **(B)** triglycerides (TG), **(C)** high-density lipoprotein cholesterol (HDL-C), **(D)** low-density lipoprotein cholesterol (LDL-C) and **(E)** fasting blood glucose (FBG). The white dot denotes the group mean, while black dots indicate outliers. TG (1.667 ± 1.173 mmol/L vs. 1.447 ± 0.954 mmol/L; *p* = 0.020) and FBG (5.655 ± 1.704 mmol/L vs. 5.363 ± 1.028 mmol/L; *p* = 0.024) were significantly higher in the *H. pylori*-positive group, whereas CHOL, HDL-C and LDL-C did not differ between groups (all *p* > 0.05). *P*-values were obtained with independent-samples *t*-tests; significance threshold *p* < 0.05.

Comparison of metabolic parameters between *H. pylori*-positive and negative groups revealed that FBG (5.655 ± 1.704 mmol/L vs. 5.363 ± 1.028 mmol/L; *p* = 0.024) and triglyceride levels (1.667 ± 1.173 mmol/L vs. 1.447 ± 0.954 mmol/L; *p* = 0.020) were both significantly higher in the positive group, whereas CHOL, HDL-C and LDL-C showed no differences between groups (all *p* > 0.05) (Figure 1).

Logistic regression analysis adjusted for multiple influencing factors revealed that *H. pylori* infection was significantly associated with abnormal FBG levels (OR = 2.102, *p* = 0.003), but not with elevated TG (*p* > 0.05). BMI emerged as a shared risk factor for FBG and TG abnormalities, with a stronger influence on TG levels (OR = 1.223, *p* < 0.01). Additionally, advancing age was only associated with blood glucose dysregulation, whereas it had no significant impact on TG levels. In terms of gender, females were more likely to develop abnormal glucose levels, but sex showed no significant association with TG abnormalities. These findings suggest that *H. pylori* infection may play a more prominent role in glucose metabolism, while BMI exerts a greater influence on lipid metabolism (Table 2).

**TABLE 2:** Binary logistic regression models incorporating multiple influencing factors for abnormal fasting blood glucose and triglyceride levels.

Clinical variables	FBG		TG	
	OR (95% CI)	*P*-value	OR (95% CI)	*P-* value
Sex	0.477 (0.281, 0.811)	0.006	0.702 (0.453, 1.086)	0.112
Age	1.051 (1.025, 1.078)	< 0.001	1.008 (0.989, 1.027)	0.428
*H. pylori*	2.102 (1.290, 3.424)	0.003	1.373 (0.916, 2.057)	0.125
Smoking	0.667 (0.309, 1.44)	0.303	0.848 (0.453, 1.588)	0.607
Alcohol drinking	0.721 (0.286, 1.820)	0.489	1.216 (0.582, 2.541)	0.603
Hypertension	1.325 (0.788, 2.228)	0.289	1.075 (0.690, 1.674)	0.750
CHD	0.587 (0.158, 2.187)	0.428	1.147 (0.390, 3.377)	0.803
CI	1.253 (0.441, 3.559)	0.672	0.610 (0.189, 1.973)	0.409
BMI	1.114 (1.026, 1.210)	0.010	1.223 (1.143, 1.309)	< 0.001

*H. pylori* infection significantly increased the risk of FBG abnormality in the overweight/obese population with BMI ≥ 24 (OR = 3.187, 95% CI: 1.586–6.404), whereas there was no significant effect of *H. pylori* in the normal-weight population with BMI < 24 (OR = 1.415, *p* = 0.330); a sex-protective effect (lower risk for females) was present only in the normal BMI group (Table 3).

**TABLE 3:** Logistic regression results analyzing the risk of *H. pylori* infection and fasting blood glucose (FBG) abnormalities stratified by body mass index (BMI).

Variables compared by BMI groups	OR (95% CI)	*P*-value
**BMI < 24**		
*H. pylori*	1.415 (0.704,2.847)	0.330
Sex	0.309 (0.157,0.606)	0.001
Age	1.059 (1.027,1.092)	< 0.001
**BMI ≥ 24**		
*H. pylori*	3.187 (1.586,6.404)	0.001
Sex	0.977 (0.481,2.034)	0.989
Age	1.036 (1.003,1.071)	0.034

**FIGURE 2:**
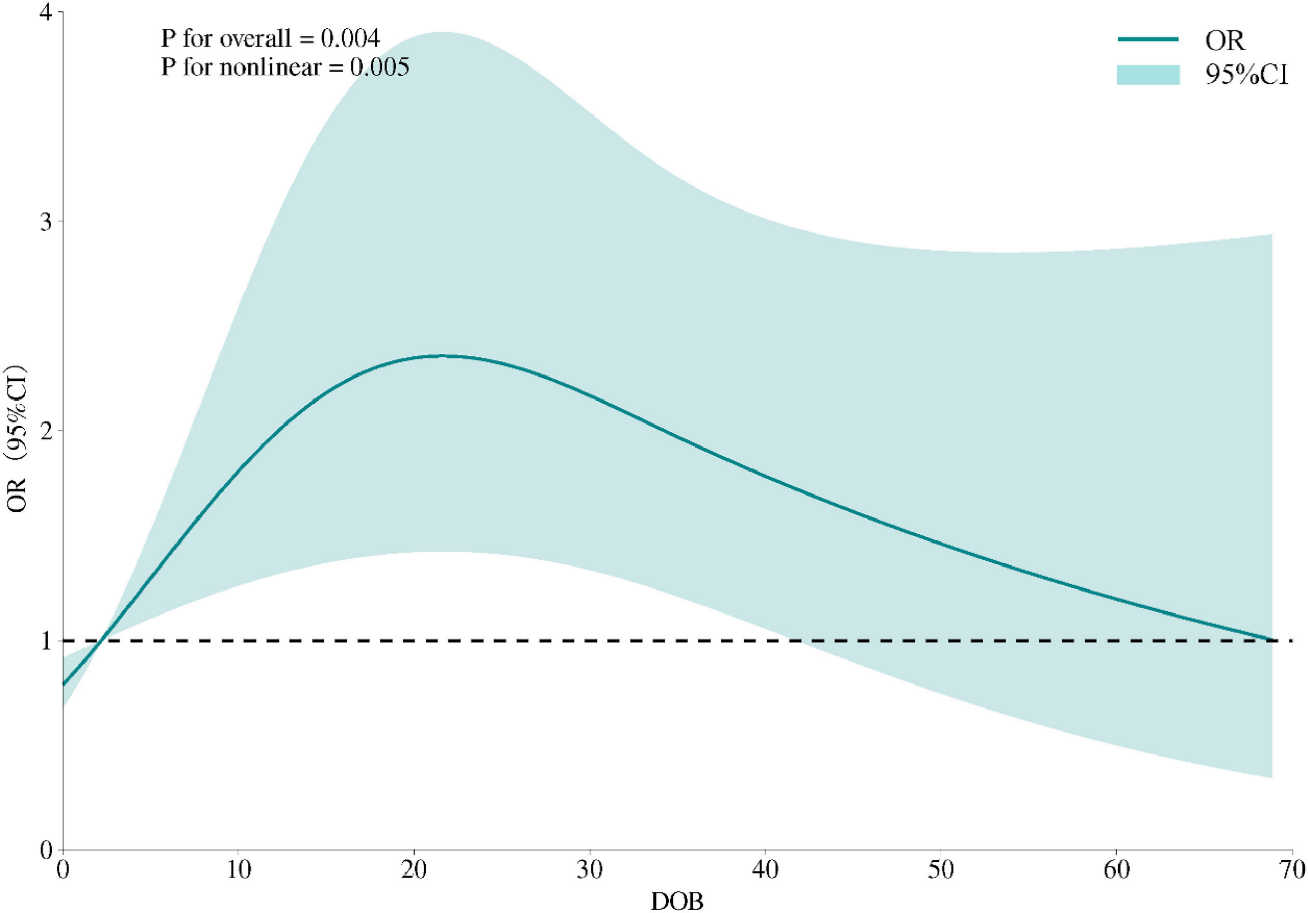
Restricted cubic spline model of the association between DOB and odds of FBG abnormality. The solid curve represents adjusted ORs, shaded area the 95% CI; knots placed at the 5th, 50th and 95th percentiles; non-linearity tested by likelihood-ratio (*p* < 0.01).

By fitting restricted cubic splines (RCS), we further explored the relationship between DOB and FBG. We predicted that the highest risk occurred when the bacterial load was approximately 20. This suggests that the bacterial load within a specific range may affect glucose regulation, the results of which are depicted in Figure 2.

**FIGURE 3:**
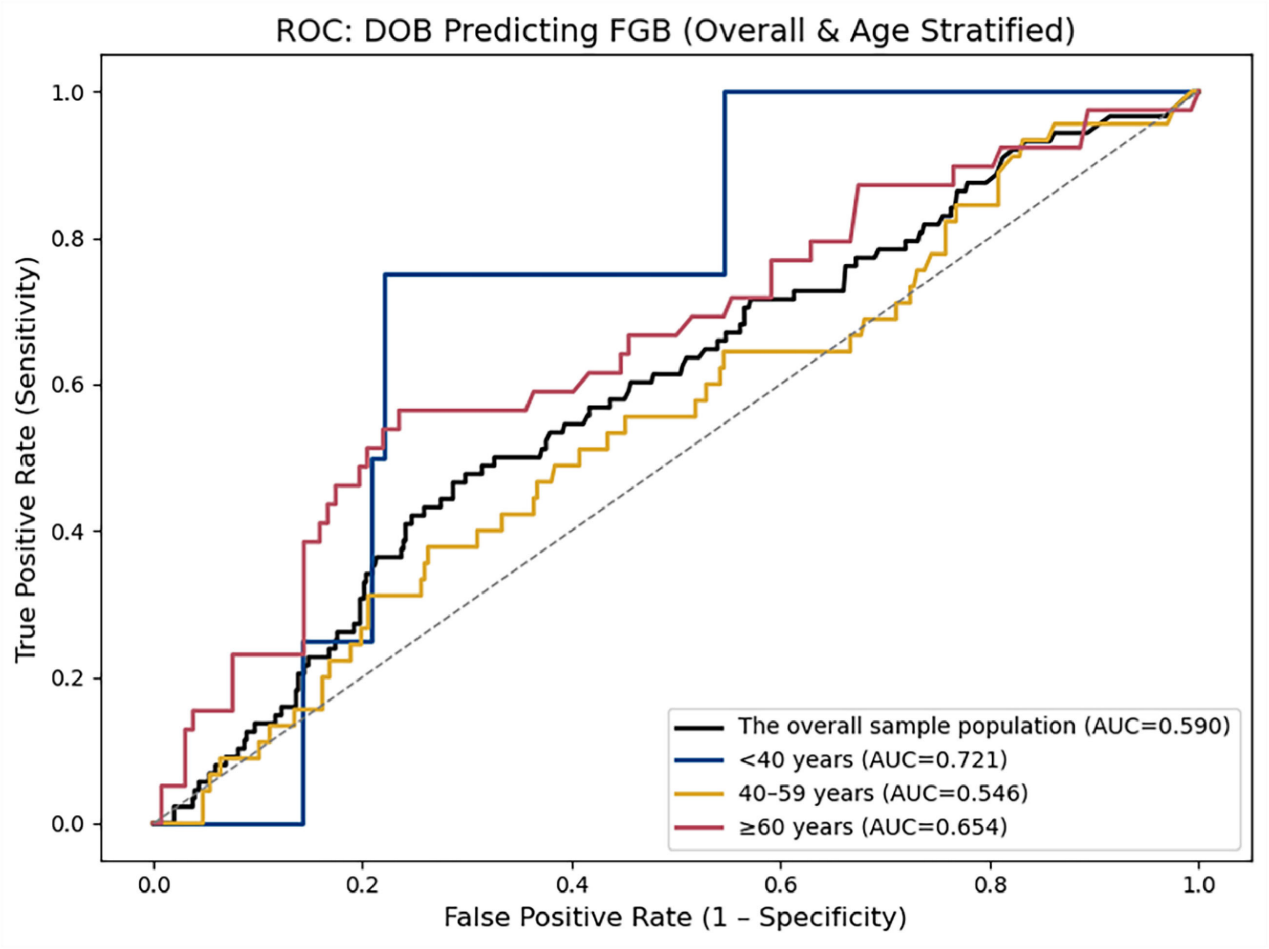
ROC curves depict the discriminatory ability of DOB in the overall cohort (black line; AUC = 0.590) and three age strata: < 40 years (blue line; AUC = 0.721), 40–59 years (gold line; AUC = 0.546), and ≥ 60 years (red line; AUC = 0.654). The diagonal dashed line indicates no-discrimination (AUC = 0.5).

Age-stratified predictive performance of DOB for abnormal fasting blood glucose (Figure 3 and Table 4).

**TABLE 4:** Predictive ability of delta-over-baseline (DOB) for fasting blood glucose (FBG) in the overall sample and across different age groups.

Population	AUC	95% CI	SE	*P*-value	Cut-off value	Sensitivity	Specificity	Youden
Overall	0.590	0.524–0.652	0.032	0.005	4.98	0.466	0.713	0.179
< 40 years	0.721	0.469–0.875	0.102	0.030	19.25	0.750	0.779	0.529
40 < 60 years	0.546	0.455–0.636	0.047	0.323	9.33	0.378	0.737	0.115
≥ 60 years	0.654	0.543–0.753	0.053	0.004	4.430	0.564	0.765	0.329

## 4 Discussion

This study explored the association between *H. pylori* infection and disorders of blood glucose and lipid metabolism, as well as the potential underlying mechanisms. Our findings demonstrated that the proportion of males, smoking, and alcohol consumption were significantly higher in the *H. pylori*-positive group than in the negative group, suggesting that lifestyle factors may influence infection risk, possibly via oral–oral transmission. Importantly, individuals with *H. pylori* infection had significantly elevated fasting blood glucose and triglyceride levels. Multivariate regression analysis further confirmed that *H. pylori* infection was significantly associated with elevated blood glucose levels. (OR = 2.102, *p* = 0.003), while BMI was independently associated with elevated TG levels (OR = 1.223, *p* < 0.001).

Several mechanisms have been proposed through which *H. pylori* may disrupt host metabolic homeostasis:

   1.  Chronic inflammation induced by infection can promote the release of pro-inflammatory cytokines such as IL-6 and TNF-α, leading to insulin resistance (IR), hepatic very-low-density lipoprotein (VLDL) overproduction (7), and suppression of lipoprotein lipase (LPL) activity, thereby impairing TG clearance (8).
   2.  Abnormal gastrin secretion resulting from infection may chronically activate the PI3K/Akt pathway, promoting β-cell apoptosis and impairing islet function, while also disrupting the gut–islet axis and attenuating the glycemic regulatory role of glucagon-like peptide-1 (GLP-1) (9, 10).
   3.  Altered bile acid metabolism, due to urease-mediated gastric acid suppression and dysbiosis, may reduce secondary bile acid synthesis and interfere with farnesoid X receptor (FXR)-mediated lipid regulation (11, 12).
   4.  Mucosal injury and vagal nerve activation, along with reduced ghrelin secretion, may suppress fatty acid oxidation and exacerbate insulin resistance (13–15).

Clinical studies have shown that eradication of *H. pylori* may improve insulin sensitivity and reduce fasting TG levels (16, 17), although findings remain inconsistent. Therefore, future studies incorporating metabolomics and single-cell analysis are needed to elucidate the molecular mechanisms of *H. pylori* virulence factors (e.g., CagA) in metabolic regulation.

Our results align with previous studies indicating a positive association between *H. pylori* infection and elevated blood glucose and TG levels. As early as 1989, Simon et al. (18) reported a higher *H. pylori* infection rate in diabetic patients than in non-diabetics (62% vs. 21%), albeit using rapid urease testing and without adjusting for age. A prospective cohort study conducted by Jeon et al. (19) involving 782 Latino individuals was the first to demonstrate that *Helicobacter pylori* (*H. pylori*) infection increases the incidence of diabetes. In addition, studies by Kim et al. (21) have shown that *H. pylori* eradication can improve HbA1c levels and metabolic abnormalities in patients with type 2 diabetes mellitus (T2DM) (20, 22). However, numerous other studies have failed to establish a significant association between *H. pylori* infection and glycemic status. For instance, Nawaz et al. (23) reported no significant difference in *H. pylori* seroprevalence between diabetic patients and non-diabetic controls. Similarly, a study primarily involving Russian and Ukrainian populations found no significant difference in *H. pylori* prevalence between individuals with T2DM and healthy controls (24), consistent with findings from several other countries (25, 26). Potentially due to: (1). Population heterogeneity (e.g., differences in ethnicity or dietary patterns); (2). Variation in diagnostic methods—^13^C-urea breath test (UBT) with DOB quantification more accurately reflects active infection compared to serology or rapid urease testing; (3). Inconsistent control of confounding factors. Notably, our ROC curve analysis showed that DOB values had statistically significant associations with blood glucose abnormalities (*p* < 0.05), despite AUC below the clinical diagnostic threshold. This suggests that while DOB may not serve as a standalone diagnostic marker, it could function as a potential risk indicator for metabolic disturbances. Furthermore, the continuous quantitative nature of the DOB value introduces the possibility of a dose–response relationship between infection severity and metabolic dysfunction, which warrants further investigation.

The innovation of this study is the joint DOB values to analyze the relationship between *H. pylori* infection and metabolic indicators, and to explore the mechanisms by which *H. pylori* infection may affect glycolipid metabolism from a multidimensional perspective. Nonetheless, several limitations should be acknowledged:

   1.  This was a single-center, cross-sectional study, which may introduce selection bias and precludes causal inference;
   2.  We did not perform *H. pylori* strain typing (e.g., CagA±);
   3.  Direct evidence on gut microbiota composition and insulin resistance was lacking.
   4.  We lacked data on diet, medication use, physical activity, and socioeconomic status, which may confound the observed associations and should be collected in future prospective studies.
   5.  Although we discuss inflammation and incretin dysregulation pathways, our study did not measure cytokines, GLP-1, or other biomarkers. This limits mechanistic inference and should be addressed in future work.

Future studies should adopt a prospective cohort design to assess whether *H. pylori* eradication improves metabolic parameters. In addition, metagenomic and animal model approaches could help clarify how microbial interactions and specific virulence factors modulate lipid metabolism at the molecular level.

## 5 Conclusion

Our cross-sectional analysis reveals that *H. pylori* infection is associated with elevated fasting blood glucose and triglyceride levels, and that the ^13^C-UBT DOB value exhibits modest discriminatory ability for glycemic abnormality (overall AUC = 0.590). Notably, age-stratified ROC analysis demonstrated that DOB’s discriminatory ability was substantially higher in participants under 40 years (AUC = 0.721), suggesting that its utility may be greatest in younger adults. Although BMI demonstrated stronger predictive power for lipid abnormalities, the DOB value may still have utility as an adjunct risk-stratification marker in contexts where standard blood tests are impractical. Because this was a single-center, cross-sectional study without data on lifestyle confounders or mechanistic biomarkers, causal inferences cannot be drawn. Future prospective, multicenter cohort studies—collecting serial UBT measurements alongside detailed clinical (e.g., diet, medications, exercise, socioeconomic status) and mechanistic (e.g., cytokines, GLP-1) data—are needed to validate and expand upon these findings.

## Data Availability

The raw data supporting the conclusions of this article will be made available by the authors, without undue reservation.
